# Oropharyngeal swab sampling for PRRSV detection in large-scale pig farms: a convenient and reliable method for mass sampling

**DOI:** 10.1186/s40813-024-00392-8

**Published:** 2024-10-10

**Authors:** Mingyu Fan, Yang Li, Zhiqiang Hu, Lujie Bian, Weisheng Wu, Wei Liu, Meng Li, Xinglong wang, Jing Ren, Lili Wu, Xiaowen Li

**Affiliations:** 1 Shandong Engineering Research Center of Pig and Poultry Health Breeding and Important Infectious Disease Purification, Shandong New Hope Liuhe Group Co., Ltd., Qingdao, 266100 Shandong China; 2 Shandong Swine-Health-Station Agriculture and Animal Husbandry Technology Co., Ltd., No.1288 Sanba East Road, Changhe Street, Tianqu New District, Dezhou, 253000 Shandong China; 3 Shandong Swine Health Data and Intelligent Monitoring Project Laboratory, Dezhou University, Dezhou, China; 4 College of Animal Science, Xichang University, No.1, Xuefu Road, Anning Town, Xichang, 615013 Sichuan Province China; 5 College of Veterinary Medicine, Northwest A&F University, Yangling, China

**Keywords:** PRRSV, Oropharyngeal swab, Individual sampling, qPCR

## Abstract

**Background:**

Porcine reproductive and respiratory syndrome virus (PRRSV) has significant productivity and economic impacts in swine herds. Accurately determining the PRRSV status at the herd level is crucial for producers and veterinarians to implement strategies to control and eliminate the virus from infected herds. This study collected oropharyngeal swabs (OSs), nasal swabs (NSs), oral fluid swabs (OFs), rectal swabs (RSs), and serum samples continuously from PRRSV challenged pigs under experimental conditions and growing pigs under field conditions. Additionally, OSs and serum samples were collected from individual sows from 50 large-scale breeding farms, and the collection of OSs does not require the sows to be restrained. Ct values of PRRSV were detected in all samples using real-time reverse transcriptase-polymerase chain reaction (RT-qPCR).

**Results:**

In PRRSV challenged pigs, OSs showed a higher PRRSV-positive rate until the end of the observation period. The Ct values of OSs were significantly lower than those of NSs, OFs, and RSs at 2, 8, 12, 14 and 20 days post-challenge (DPC) (*P* < 0.05). For growing pigs, the positivity rate of PRRSV in OSs was higher than that in other sample types at 30, 70, and 110 days of age. In sows, 24,718 OSs and 6259 serum samples were collected, with PRRSV-positive rate in OSs (9.4%) being significantly higher than in serum (4.1%) (*P* < 0.05). However, the Ct values of PRRSV RNA in serum were significantly lower than those in OSs (*P* < 0.001).

**Conclusions:**

The OSs sample type yielded higher PRRSV-positive rates for longer periods compared to NSs, RSs, OFs and serum samples for PRRSV detection in infected pigs. Therefore, OSs has a good potential to be a convenient, practical, and reliable sample type for implementing mass sampling and testing of PRRSV in large-scale pig farms.

**Supplementary Information:**

The online version contains supplementary material available at 10.1186/s40813-024-00392-8.

## Background

Porcine reproductive and respiratory syndrome (PRRS) is recognized as a significant economic threat to the pig industry. The causative agent, porcine reproductive and respiratory syndrome virus (PRRSV), is a positive-stranded enveloped RNA virus classified under the genus *Betaarterivirus*, family *Arteriviridae*, and order *Nidovirales* [1]. The PRRSV has two known variants: *Betaarterivirus* suid 1, previously known as PRRSV-1, and suid 2, previously known as PRRSV-2 [2]. PRRSV impairs the reproductive capabilities of sows and diminishes the growth and feed conversion rates, and has a significant impact on mortality in fattening pigs [3, 4]. It targets the immune system, weakening defenses against pathogens, thereby increasing susceptibility to secondary infections and intensifying the disease [5]. Consequently, producers and veterinarians strive to manage and potentially eradicate PRRSV in large-scale pig farms.

Determining the herd-level PRRSV status is crucial for effective disease prevention and control. Established population-based sampling methods, such as oral fluids, family oral fluids, processing fluids, and tongue tip fluids, facilitate monitoring at various ages stages [6–9]. Although these methods increase herd sensitivity by testing more animals, they face challenges in detecting low-level PRRSV presence [10–12]. Therefore, obtaining precise individual PRRS data is essential for specific diagnosis and epidemic monitoring, which supports implementing tailored prevention and control measures, including introducing PRRSV-negative gilts and eradicating PRRS from breeding herds [13]. Although serum samples and tonsil scrapings are common for acquiring individual infection data, they require restraining pigs and are labor-intensive, time-consuming, and relatively invasive [14].

Tonsil scraping has proven effective in isolating PRRSV from long-term infected pigs [15, 16], and has shown to yield more positive results over extended periods compared to oral fluid, NSs, and environmental samples [17]. In China, our team has successfully eradicated African swine fever virus (ASFV) from pig herds using a “test-removal method” based on rapid ASFV testing by an oropharyngeal swab sampling tool (OST), which allows rapid, individual sample collection without restraining the sows and minimizes cross-contamination [18]. The collected OSs fluid, containing tonsil exudate and oral fluid, also shows promise as a sample type for PRRSV detection. The aim of this study is to evaluate the use of individual pig OS for determining PRRSV infection status. We conducted animal challenge studies and field condition sampling method evaluations to assess the effectiveness of the OST in detecting PRRSV at the individual level.

## Materials and methods

### Detection of PRRSV in different types of samples from challenged pigs

Eight healthy piglets were sourced from a PRRSV-free herd and weaned at three weeks of age. All animals were confirmed negative for PRRSV, CSFV, PRV, SIV, and PCV2 infection as previously described [19]. At four weeks of age, piglets were randomly divided into two groups and individually housed in isolation units during the study period. The challenge group (n = 6) received an intramuscular inoculation of 2 × 10^5.0^ TCID_50_ of PRRSV XM-2020 strain (GenBank accession number: MZ160905.1). The control group (n = 2) received an intramuscular injection of 2 ml phosphate buffered saline (PBS). Rectal temperatures were recorded daily. At each sampling time (Table 1), samples of OSs, NSs, RSs, OFs, and serum were collected from each pig. All animals were euthanized at 28 DPC.

**Table 1:** PRRSV detection results in different types of samples from challenged pigs

Sample type	Proportion of positive samples at the indicated days post-challenge																	
	0	2	3	4	5	6	7	8	10	12	14	16	18	20	22	24	26	28
OSs	0/6	6/6	6/6	6/6	6/6	6/6	6/6	6/6	6/6	6/6	6/6	6/6	5/6	6/6	6/6	6/6	5/5	5/5
NSs	0/6	1/6	4/6	4/6	6/6	6/6	4/6	5/6	1/6	0/6	0/6	2/6	0/6	1/6	2/6	1/6	0/5	0/5
RSs	0/6	2/6	6/6	6/6	3/6	4/6	4/6	5/6	2/6	4/6	2/6	2/6	1/6	1/6	3/6	1/6	0/5	0/5
OFs	0/6	4/6	6/6	5/6	5/6	4/6	4/6	6/6	5/6	4/6	3/6	3/6	2/6	3/6	2/6	2/6	0/5	0/5
Serum	0/6	6/6	–	–	–	–	6/6	–	–	–	6/6	–	–	6/6	–	–	–	0/5

All samples were collected for the first time at 0 DPC. OS, NS, RS and OFs were collected once daily at 2–8 DPC and every other day at 10–28 DPC. Serum samples were collected at 2, 7, 14, 20, 28 DPC

*DPC* days post-challenge; *OSs* oropharyngeal swabs; *NSs* nasal swabs; *RSs* rectal swabs; *OFs* oral fluid swabs

### Detection of PRRSV in growing pigs under field conditions

A fattening farm in Shandong Province, China, with a single-sourced wean-to-finish, all-in/all-out pig flow (herd size: 6000) participated in this study. The source breeding farm of weaned piglets had experienced PRRSV outbreaks within the last six months. All piglets were vaccinated with 1 dose of modified live PRRSV vaccine at 14 days of age. Twenty pigs, marked with special ear tags, were randomly selected at 30 days of age. Subsequently, the pigs were sampled at 30, 70, and 110 days of age. At each sampling time, samples of OSs, NSs, RSs, OFs and serum were collected from each pig.

### Detection of PRRSV in sows under field conditions

In 2022, clinical samples comprising OSs and serum from sows were collected across 50 large-scale breeding farms in China (herd sizes between 3000 and 13,500). All farms were assumed to be PRRSV positive. Each farm provided more than 30 unpaired OSs and serum samples. All farms followed identical PRRSV vaccination protocols, vaccinating sows four times annually with a modified live PRRSV vaccine.

### Sample collection

**Fig. 1:**
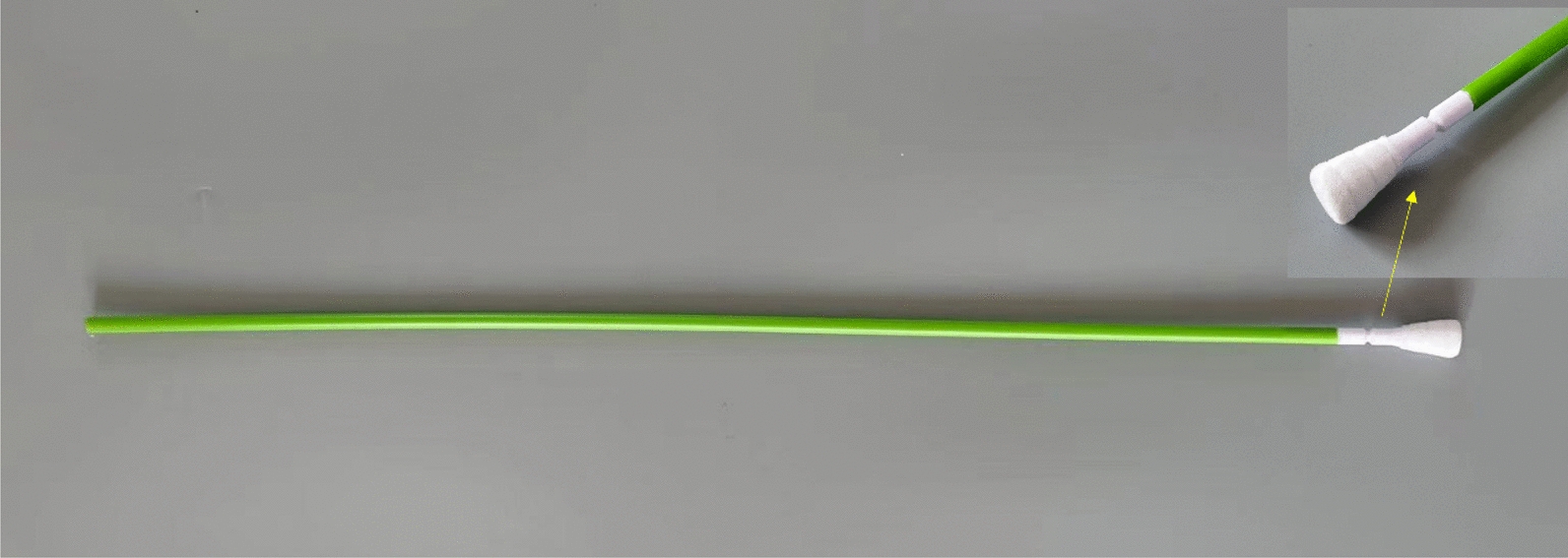
General view and local feature diagram of the OST. The handle of the OST is a plastic shaft with a length of 55 cm and the head of the OST is a pear-shaped flocked polyester fibre. Between the head and the handle is a circular groove. OST: oropharyngeal swab sampling tool

**Fig. 2:**
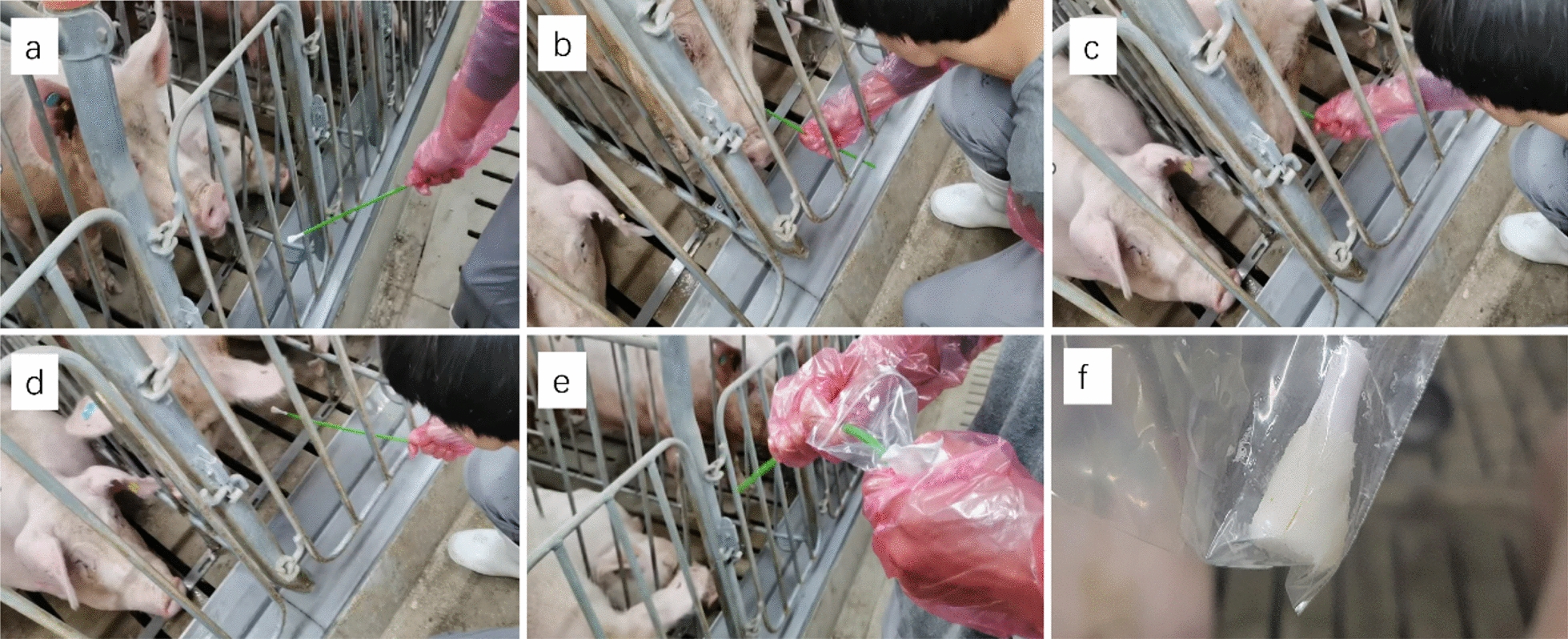
OSs fluid sampling by the OST in sow farms (See Additional File 1). Before collecting (**a**); the sow gnaws and licks the head of OST (**b**); inserting the OST into the pig’s throat (**c**); removing back the OST (**d**); breaking off the head of OST (**e**); OS fluid collection (**f**). OST: oropharyngeal swab sampling tool

For the serum sample, the pig was restrained with a nose snare, blood was drawn from the anterior vena cava, rested at room temperature for 30 min, then centrifuged at 1000 g for 2 min to separate the serum. For RSs and NSs, a short cotton swab (Haishihainuo, Qingdao, China) was inserted into a pig’s nose or rectum, then withdrawn and eluted with 1 mL saline solution, respectively. For OFs, a short cotton swab was inserted into the pig’s mouth and chewed until thoroughly moistened, then eluted with 1 mL saline solution. The OSs of the sows were collected in individual stalls without any restraint, as previously described [14]. The OST, featuring a pear-shaped flock head and a long plastic shaft (Zhixing Animal Husbandry Technology, Qingdao, China), was used for sample collection (Fig. 1). The sow actively gnawed and licked the OST, which was then moved back and forth in the pig’s throat and the tonsil area with an upwards angle. The qualified sample was defined as viscous and mucous-like. Then, the head with oral fluid and tonsil exudate was broken off and eluted into a sealed bag with 2 mL saline solution (Fig. 2). The eluent was transferred into a plastic tube. However, the group-housed pigs must be restrained with a nose snare before the OSs were collected.

### RT-qPCR

Swab eluents and serum were vortexed and centrifuged at 5000 g for 1 min. Total RNA (200 μL) from each sample was extracted using the TRIzol protocol (TaKaRa, Tokyo, Japan). Then, the extracted RNAs were tested for PRRSV using RT-qPCR test kits (Jiazhi Biotech, Qingdao, China), and the detection limit of qPCR assay is 2.5 copies/uL of the PRRSV genome. A sample was deemed positive if the Ct value was ≤ 40 and the curve showed a specific exponential look.

### Statistical analysis

Statistical analyses were conducted using SPSS Statistics version 22. Chi-squared tests with Bonferroni adjustments were used to compare PRRSV-positive proportions across different samples. In challenge pigs and growing pigs, the PRRSV mean Ct values of different positive samples on the indicated sampling day were analyzed using one-way ANOVA, with a *P* value < 0.05 considered significant. For sow samples, positive rates were expressed as absolute and relative frequencies (%) with a 95% confidence interval. Mean Ct values of PRRSV testing in OSs and serum samples were analyzed using independent samples t-tests, with a *P* value < 0.05 considered significant.

## Results

### PRRSV detection in challenged pigs

**Fig. 3:**
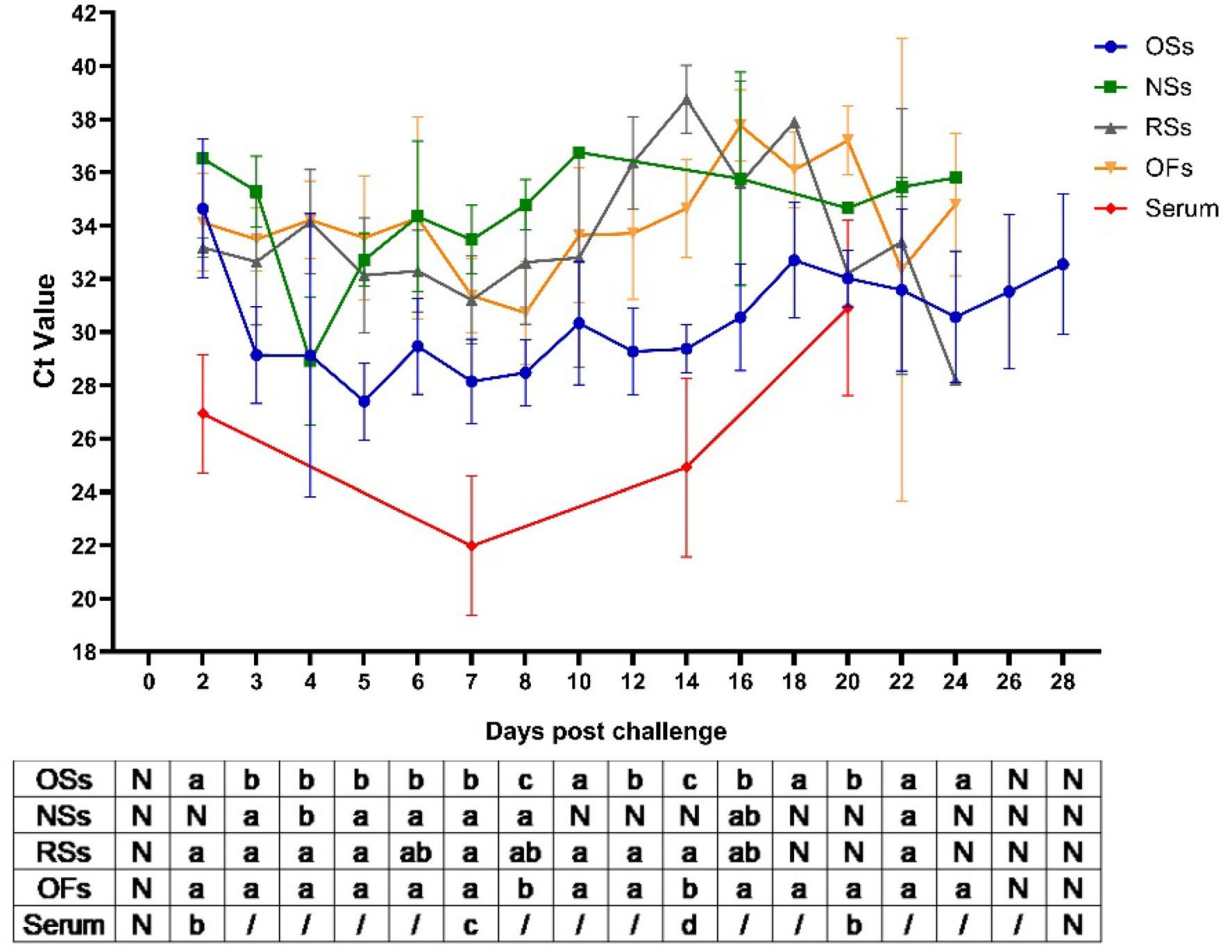
Dynamics of Ct values of PRRSV-positive samples from challenged pigs. The Ct values with letters a, b and c are with significant differences between different types of positive samples. Different letters indicate significant statistical differences (*P* < 0.05), and the same letter indicates no significant statistical differences (*P* > 0.05). The letter ‘N’ indicates there were not enough PRRSV positive samples for one-way ANOVA of Ct values

All PRRSV-challenged piglets exhibited symptoms such as high fever, depression, anorexia, respiratory distress, and decreased weight gain following PRRSV exposure, with one piglet succumbing at 25 DPC. Serum samples from these pigs consistently tested positive for PRRSV up to 20 DPC, showing significantly lower Ct values compared to other sample types at 2, 7 and 14 DPC (*P* < 0.05), as depicted in Table 1 and Fig. 3. Additionally, Ct values of OSs were significantly lower (*P* < 0.05) than those of NSs, RSs and OFs at 2–8, 12, 14, 20 DPC (Fig. 3). By 28 DPC, only OSs tested positive, indicating a more sustained detection capability throughout the study period (Table 1).

### PRRSV detection in growing pigs under field conditions

At 30 days of age, 15% of the pigs (3 out of 20) tested positive for PRRSV via OSs, while only 5% (1 out of 20) were positive by serum and OFs samples. Neither NS nor RS samples tested positive at this stage. By 70 days of age, 100% of OSs (20 out of 20) were positive, followed by 50% for OFs and serum, 20% for RS, and 15% for NS. The number of samples decreased as two pigs died at 74 and 89 days of age. At 110 days of age, the highest positivity rate was observed in OSs (55.6%), with lower rates in serum samples (33.3%), OFs (27.8%), RSs (22.2%), and NSs (11.1%) samples. OSs consistently showed higher detection rates throughout the study, with significant differences noted at 70 days. There were no significant differences in Ct values among sample types at each sampling age (*P* > 0.05) (Table 2).

**Table 2:** Detection of PRRSV in growing pigs under field condition

Sample types	30 days of age		70 days of age		110 days of age	
	Positivity rate	Ct value Mean ± SD	Positivity rate	Ct value Mean ± SD	Positivity rate	Ct value Mean ± SD
OSs	3/20 15.0%^A^	35.5 ± 1.3	20/20 100%^B^	34.4 ± 2.0^a^	10/18 55.6%^B^	33.9 ± 1.8^a^
NSs	0/20 0.0%^A^	–	3/20 15.0%^A^	33.2 ± 2.0^a^	2/18 11.1%^A^	36.1 ± 2.5^a^
RSs	0/20 0.0%^A^	–	4/20 20.0%^A^	34.6 ± 1.6^a^	4/18 22.2%^AB^	37.5 ± 1.0^a^
OFs	1/20 5%^A^	36.5 ± 0	10/20 50.0%^A^	35.9 ± 2.3^a^	5/18 27.8%^AB^	35.1 ± 3.1^a^
Serum	1/20 5%^A^	35.4 ± 0	10/20 50.0%^A^	34.4 ± 2.5^a^	6/18 33.3%^AB^	31.8 ± 4.4^a^

Different letters A, B and C indicate significant differences in PRRSV-positive proportions for samples. Mean values with letters a, b and c are with significant differences between different types of positive samples. Different letters indicate significant statistical differences (*P* < 0.05). The ‘–’ indicates that the samples were PRRSV negative

*Oss* oropharyngeal swabs; *NSs* nasal swabs; *RSs* rectal swabs; *OFs* oral fluid swabs

### PRRSV detection in sows from large-scale breeding farms

**Fig. 4:**
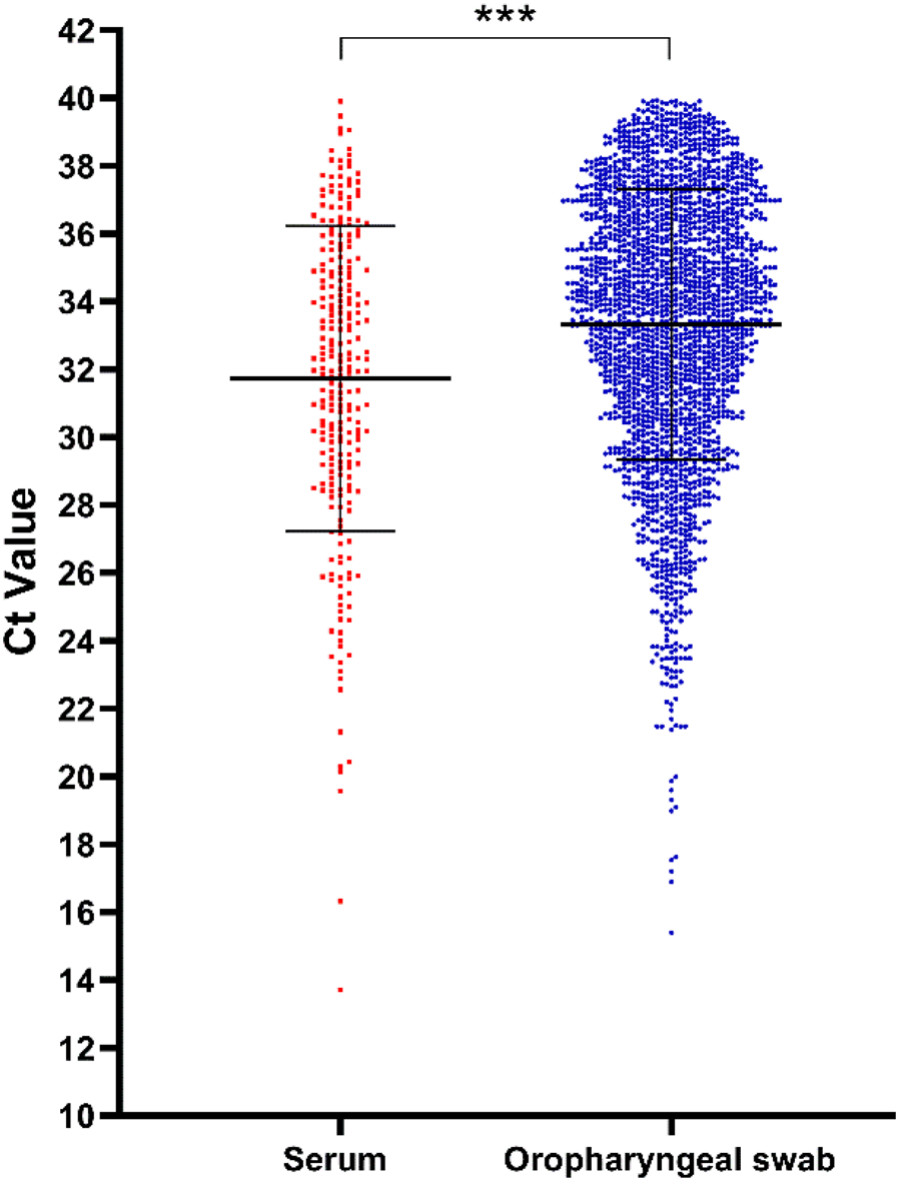
The Ct value of PRRSV-positive OSs and serum samples. ***P < 0.001

**Fig. 5:**
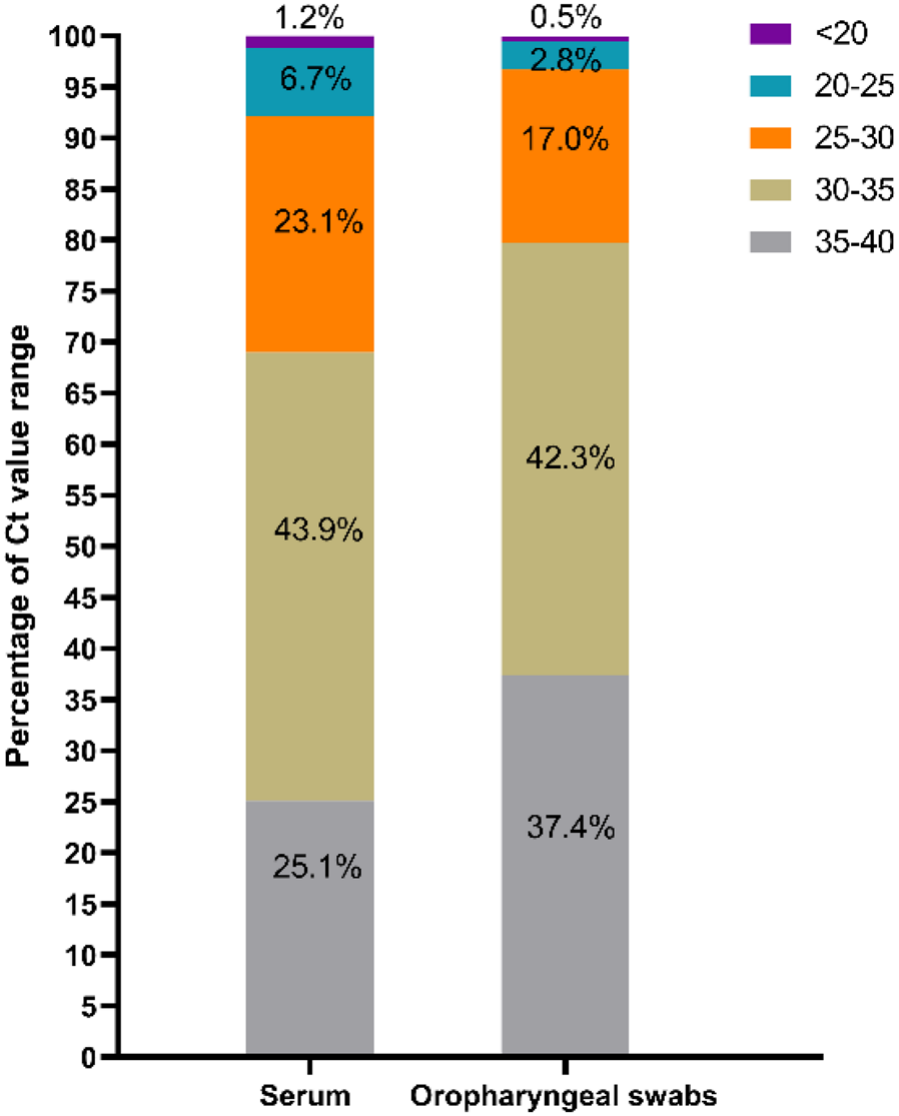
Distribution of Ct values of PRRSV-positive samples

In 2022, a total of 24,718 OSs and 6259 serum samples were collected from 50 large-scale breeding farms across China. Of these, 32 farms tested positive for PRRSV via serum (64.0%, 95% CI 50.2%–77.8%), while an impressive 49 farms (98%) were positive via OSs (98%, 95% CI 94.0%–100.0%), as detailed in Table 3. The positivity rate for OSs was 9.4%, significantly higher than the 4.1% for serum samples (*P* < 0.05) (Table 3). However, Ct values for PRRSV-positive serum samples were notably lower than those for OSs (*P* < 0.001), indicating higher viral loads in the serum, as illustrated in Fig. 4. Additionally, Ct values were categorized into five ranges: < 20, 20–25, 25–30, 30–35, and 35–40. Notably, serum samples had a higher proportion of Ct values in the range of 20–30 compared to OSs, which had a higher proportion in the 35–40 range. Both sample types showed similar distributions in the 30–35 range, as depicted in Fig. 5.

**Table 3:** PRRSV positivity rates in serum and oropharyngeal swab samples from sows under field condition

Sample type	Tested farm	Positive farm※	Positivity rate at the farm level	Tested sample	Positive sample	Positivity rate at the sample level
Serum	50	32	64% CI 50.2%–77.8%	6259	255	4.1%^a^ CI 3.6%–4.6%
Oropharyngeal swabs	50	49	98% CI 94.0%–100.0%	24,718	2324	9.4%^b^ CI 9.0%–9.8%

CI, 95% confidence interval for the sample positivity rate. Different letters indicate significant differences in PRRSV-positive proportions for samples (*P* < 0.05). ※: “Positive farm” mean the farm which the counterpart samples tested positive for PRRSV

## Discussion

In PRRSV-challenged pigs, OSs demonstrated a higher and more sustained detection rate and consistently lower Ct values compared to other peripheral swab samples throughout the observation period. The development of viremia and the distribution of susceptible macrophages enable PRRSV to be shed through various routes including saliva, nasal secretions, and other bodily excretions. Despite the potential for using these excretions for PRRSV detection, the duration of shedding is typically short and transient [20]. This trend was similarly observed in growing pigs over a prolonged observation period. During the early stages (2, 7, 14, 20 DPC), all serum samples tested positive for PRRSV. However, by 28 DPC, PRRSV was undetectable in serum samples. In field conditions, the detection rate of PRRSV in serum samples was consistently lower than in OSs at 70 and 110 days of age. In adult pigs, viremia may be confined to only few weeks [21, 22], yet PRRSV can persist in the tonsil for 150–250 days [15, 16, 23], underscoring the effectiveness of OSs for long-term PRRSV detection.

In this study, we used the OST with pear-shaped rod head to collect OSs from 50 large breeding farms. At the sample level, OSs detection rates were more than double those of serum. Furthermore, while 17 sow farms tested negative for PRRSV in serum samples, their OSs were positive, highlighting the sensitivity of OSs. Additionally, the collection of mass serum samples poses a high biosecurity risk in sow herds, particularly with the prevalence of ASFV in China [24]. As OSs sampling does not require the sow to be restrained compared to serum, exposure of sampling personnel and tools to pigs and the environment contaminated with pig secretions is reduced. The acceptability of OSs collection was evident in breeding farms, with significantly more OSs collected compared to serum. Although a higher PRRSV-positive rate of OSs was detected in sows, viral loads were significantly lower than those in serum samples. And Ct values of serum and OSs were mainly clustered in the range of 30–35, with a greater proportion of OSs in the range of 35–40. A possible explanation could be that the viremic phase of infection is followed by virus confinement in secondary lymphoid tissues, leading to lower viral replication [20]. Additionally, OSs were diluted in a larger volume of media compared to serum, which could influence Ct value comparisons. The results of this study were inconsistent with those reported by Li et al. [14], in which the PRRSV CT value of tonsil-oral scraping samples similar to OSs was lower than that of serum samples in acutely infected sows. This discrepancy may be explained by differences in the number of samples as well as differences in the stage of PRRSV infection in the sows from which the samples were originated, and OSs and serum samples covered more sow herds in this study. On the other hand, the tonsil-oral scraping tool is manually assembled from the artificial insemination rod, rubber thimble and cotton gauze and are suitable for small-scale use [14], while OST has been industrially produced and marketed, providing the basis for its widespread use on breeding farms.

In breeding farms, processing fluids, family oral fluids, and tongue tips are population-based surveillance samples collected from suckling pigs and have been described for the surveillance of PRRSV in sows and piglet populations at processing and weaning [7–9], which aids in managing PRRSV control and elimination efforts [25]. It has been reported that population-based methods increase herd sensitivity without increasing cost, time and labor [9, 26], compared to bleeding a subset of animals. However, these population-based sampling schemes commonly used for PRRSV monitoring do not cover all pig production phases, especially gestating sows. Obviously, the OSs were individual samples collected from sows and were suitable for estimating farm-level prevalence. In addition, considering the costs, it is feasible to pool several OSs for PRRSV surveillance in sows. Dee et al. [27] achieved the elimination of PRRSV using a test and removal process, and the authors believed that tonsillar biopsies could serve as an additional diagnostic tool to improve elimination efficiency. Consistent with this, our study proved that the OSs containing tonsil exudate can be used as an additional diagnostic tool for PRRSV elimination.

In this study, OSs and serum samples were observed to have better detection rates than RSs, NSs and OFs as the disease progressed in PRRSV challenge and growing pigs. We speculate that the use of OSs and serum may be advantageous in the detection of PRRSV in large-scale breeding farms. Therefore, this study compared the detection efficiency of PRRSV in OSs and serum samples in sows from 50 breeding farms over one year and further confirmed that PRRSV was detected at higher detection rate in OSs. A limitation of this study was that the study design did not allow calculation of sensitivity or specificity for serum and OSs, as samples were not taken from the same sow for comparison. However, one of the aims of this work was to investigate the method of sampling OSs that can be used on a large scale in sow herds, as sampling OSs is more convenient and acceptable than serum.

## Conclusions

This is the first report of using OSs for the detection of PRRSV in large-scale breeding farms under field conditions. The study demonstrated that OSs yielded higher PRRSV-positive rates for longer periods compared to NSs, RSs, OFs, and serum samples for PRRSV detection under both experimental and field conditions. The utilization of OST for OSs sampling shows promise as a convenient, practical, and reliable method for mass individual sampling and testing of PRRSV in large-scale pig farms, thereby enhancing the implementation of effective disease management and control measures.

## Supplementary Information

Additional file1.

## Data Availability

The dataset generated in this study is available from the corresponding author on reasonable request.

## Abbreviations

ASFV: African swine fever virus
CSFV: Classical swine fever virus
DPC: Days post-challenge
NSs: Nasal swabs
OSs: Oropharyngeal swabs
OST: Oropharyngeal swab sampling tool
OFs: Oral fluid swabs
PBS: Phosphate buffered saline
PCV2: Porcine circovirus type 2
PRRSV: Porcine reproductive and respiratory syndrome virus
PRV: Pseudorabies virus
RSs: Rectal swabs
RT-qPCR: Real-time reverse transcriptase-polymerase chain reaction
SIV: Swine influenza virus
TCID_50_: 50% Tissue culture infectious doses
